# Hospital Sunday Fund

**Published:** 1902-02-08

**Authors:** 


### HOSPITAL SUNDAY FUND.

The sum collected last year (ending October 31st, 1901) amounted to £54,731 19s. 3d., being £2,738 4s. 9d. in excess of the amount collected in 1900, and, with the exception of 1895, when the Fund exceeded £60,000, the highest amount yet recorded. The collections made on Hospital Sunday (June 16th) amounted to £36,388 6s. 4d. This is an increase of £532, and 16 congregations joined the number of those collecting for the Fund. Mr. George Herring for the third time sent £10,000; Sir Frederick L. Cook, M.P., contributed a special donation of £4,000; and Sir Savile Crossley gave his tenth donation of £500. Applications for awards were received from 200 institutions, and after three had been eliminated (two dispensaries and one convalescent home), 197 awards were recommended by the committee of distribution. Five per cent. of the total sum collected was set apart for the purchase of surgical appliances, in accordance with Law XII. This amounted to about £2,500. Tables of statistics were prepared for the special use of members, giving an analysis of the number of beds in hospitals, the cost of patients both in hospitals and at dispensaries, and the proportionate expense of management, with other detailed and valuable information. The working expenses, including rent of the offices at 18 Queen Victoria Street, E.C. were £1,801 3s. 9d. as compared with the average expenditure of £1,740 5s. 8d. during 28 previous years; the proportion being 3·292 per cent. of the gross receipts. The average percentage of working expenses for the 28 years has been 3·315.

